# Herpes Simplex Virus-1 Induced Serotonin-Associated Metabolic Pathways Correlate With Severity of Virus- and Inflammation-Associated Ocular Disease

**DOI:** 10.3389/fmicb.2022.859866

**Published:** 2022-03-22

**Authors:** Diana Marie Battaglia, Maria D. Sanchez-Pino, Charles D. Nichols, Timothy P. Foster

**Affiliations:** ^1^Department of Microbiology, Immunology, and Parasitology, Louisiana State University Health Sciences Center, New Orleans, LA, United States; ^2^Department of Interdisciplinary Oncology, Louisiana State University Health Sciences Center, New Orleans, LA, United States; ^3^Department of Genetics, Louisiana State University Health Sciences Center, New Orleans, LA, United States; ^4^The Stanley S. Scott Cancer Center, Louisiana State University Health Sciences Center, New Orleans, LA, United States; ^5^Department of Pharmacology and Experimental Therapeutics, School of Medicine, Louisiana State University Health Sciences Center, New Orleans, LA, United States; ^6^Department of Ophthalmology, Louisiana State University Health Sciences Center, New Orleans, LA, United States; ^7^The Louisiana Vaccine Center, New Orleans, LA, United States

**Keywords:** herpes, serotonin, amino acid metabolism, viral replication, herpetic keratitis and cornea, eye, drug target, pathogenesis

## Abstract

Herpes simplex virus-associated diseases are a complex interaction between cytolytic viral replication and inflammation. Within the normally avascular and immunoprivileged cornea, HSV ocular infection can result in vision-threatening immune-mediated herpetic keratitis, the leading infectious cause of corneal blindness in the industrialized world. Viral replicative processes are entirely dependent upon numerous cellular biosynthetic and metabolic pathways. Consistent with this premise, HSV infection was shown to profoundly alter gene expression associated with cellular amino acid biosynthetic pathways, including key tryptophan metabolism genes. The essential amino acid tryptophan is crucial for pathogen replication, the generation of host immune responses, and the synthesis of neurotransmitters, such as serotonin. Intriguingly, Tryptophan hydroxylase 2 (TPH2), the neuronal specific rate-limiting enzyme for serotonin synthesis, was the most significantly upregulated gene by HSV in an amino acid metabolism PCR array. Despite the well-defined effects of serotonin in the nervous system, the association of peripheral serotonin in disease-promoting inflammation has only recently begun to be elucidated. Likewise, the impact of serotonin on viral replication and ocular disease is also largely unknown. We therefore examined the effect of HSV-induced serotonin-associated synthesis and transport pathways on HSV-1 replication, as well as the correlation between HSV-induced ocular serotonin levels and disease severity. HSV infection induced expression of the critical serotonin synthesis enzymes TPH-1, TPH-2, and DOPA decarboxylase (DDC), as well as the serotonin transporter, SERT. Concordantly, HSV-infected cells upregulated serotonin synthesis and its intracellular uptake. Increased serotonin synthesis and uptake was shown to influence HSV replication. Exogenous addition of serotonin increased HSV-1 yield, while both TPH-1/2 and SERT pharmacological inhibition reduced viral yield. Congruent with these *in vitro* findings, rabbits intraocularly infected with HSV-1 exhibited significantly higher aqueous humor serotonin concentrations that positively and strongly correlated with viral load and ocular disease severity. Collectively, our findings indicate that HSV-1 promotes serotonin synthesis and cellular uptake to facilitate viral replication and consequently, serotonin’s proinflammatory effects may enhance the development of ocular disease.

## Introduction

Herpes simplex virus (HSV) type-1 is a major human pathogen and is the leading cause of infection-related vision loss (Whitley and Roizman, 2001; McCormick et al., 2021). In 2016, the WHO estimated that over 3.7 billion individuals under the age of 50 were HSV-1 carriers. In the United States alone, over 500,000 Americans have experienced ocular herpetic disease with nearly 24,000 new cases and over 58,000 recurrent episodes diagnosed annually (Liesegang, 2001; Pepose et al., 2006; Young et al., 2010; Looker et al., 2015). Following primary replication within the corneal epithelium, HSV establishes a lifelong, latent infection of innervating neurons with sporadic episodes of reactivation and recrudescent disease (Whitley and Roizman, 2001; Toma et al., 2008). HSV-associated eye disease ranges in severity from blepharitis, conjunctivitis, and dendritic keratitis to vision-threatening herpetic stromal keratitis (HSK). HSK is a chronic immune-mediated progressive corneal opacification characterized by immune cell infiltrates, angiogenesis, and corneal nerve degeneration that worsens with each successive viral reactivation (Wilhelmus et al., 1996, 2020; Liesegang, 1999; Whitley and Roizman, 2001; Divito and Hendricks, 2008; Toma et al., 2008). Despite the availability of effective anti-herpetics, current antiviral therapies do not eliminate latent HSV reservoirs and prophylactic use of antivirals only reduces recurrent ocular disease by ~45% (Barron et al., 1994; Lairson et al., 2003; Young et al., 2010). Additionally, antiviral therapies fail to prevent or resolve vision-impairing T cell-mediated immunopathology and corneal scarring (Russell et al., 1984; Doymaz and Rouse, 1992; Niemialtowski and Rouse, 1992; Banerjee et al., 2004; Osorio et al., 2004; Jaggi et al., 2018; Rajasagi and Rouse, 2019). Therefore, the influence of viral and host factors on ocular disease development remains an area of intense study.

As obligate intracellular pathogens, viral replicative processes are entirely dependent upon numerous cellular biosynthetic and metabolic pathways. Consequently, viruses often redirect normal cellular processes to provide resources essential for efficient viral replication (Maynard et al., 2010; Pei and Robertson, 2020). Of the diverse biosynthetic processes modified by viruses, amino acid metabolic pathways play vital roles not only in viral protein synthesis, but also in energy production, RNA/DNA synthesis, and host immune responses to pathogens (Prusinkiewicz and Mymryk, 2019; Purdy and Luftig, 2019; Thaker et al., 2019). L-Tryptophan (L-Trp) is one of nine essential amino acids and is the least abundant of all the dietary amino acids. In addition to its role in protein synthesis, L-Trp is a precursor for two important metabolic pathways, the kynurenine pathway (KP), and the serotonin pathway (Richard et al., 2009; Barik, 2020). Physiologically, L-Trp and its metabolites are key regulators of energy production, cellular redox state, neuronal and vascular function, wound healing, as well as innate and adaptive immunity (Richard et al., 2009; Serbecic et al., 2009; Li et al., 2012; Boccuto et al., 2013; Barik, 2020; Costantini et al., 2020; Fiore and Murray, 2021; Haq et al., 2021; Silvano et al., 2021). Importantly, induction of L-Trp metabolic pathways not only facilitates immunological tolerance and maintenance of immune privilege in the eyes, brain, and placenta, but also modulates pathogen replication through regulation of L-Trp bioavailability (Serbecic et al., 2009; Li et al., 2012; Costantini et al., 2020; Fiore and Murray, 2021; Silvano et al., 2021). Indeed, one mechanism by which interferon gamma (IFN-g) suppresses pathogen replication, including Chlamydia, Hepatitis B virus, and Parainfluenza virus, is by depleting L-Trp through activation of indoleamine-pyrrole 2,3-dioxygenase (IDO) and the initial KP rate-limiting enzyme (Carlin et al., 1989; Lepiller et al., 2015; Rabbani et al., 2016; Yoshio et al., 2016; Raniga and Liang, 2018). Conversely, IDO expression can also enhance viral replication and disease manifestations associated with many viral pathogens by suppressing cell-intrinsic type I IFN antiviral responses (Hoshi et al., 2012; Kim et al., 2016).

L-Tryptophan can also be metabolized through the serotonin pathway to serotonin (5-hydroxytryptamine; 5-HT), a ubiquitous neurotransmitter and hormone that influences a broad spectrum of physiological processes. Although best characterized for its effects in the central nervous system (CNS), the majority of serotonin exists in the periphery where it has significant roles in metabolism, cell protection, cardiovascular function, vasoconstriction, gastrointestinal peristalsis, wound healing, and inflammation (Nichols and Nichols, 2008; Kring et al., 2009). The synthesis of 5-HT from L-Trp is initiated by the rate-limiting enzyme, tryptophan hydroxylase (TPH). Two different genes encode two isoforms of TPH, with TPH-1 being predominantly expressed in the periphery and TPH-2 expression confined almost exclusively to the CNS (Walther et al., 2003; Walther and Bader, 2003; Liu et al., 2008). In mammals, serotonin exerts its effects through seven different receptor families comprised of 14 distinct subtypes (Nichols and Nichols, 2008). Most immune cells express a large and varied repertoire of these 5-HT receptors, suggesting that they possess the ability to respond to serotonin (Herr et al., 2017). Accordingly, upon antigen stimulation, 5-HT provides an accessory signal to T cells that enhances both their activation and proliferation (Leon-Ponte et al., 2007; Herr et al., 2017).

Recent studies have begun to associate 5-HT pathways and its receptors with enhancement of viral infection, replication, and disease progression. Several viruses, including JC polyomavirus, Hepatitis C virus, Ebola, and Marburg viruses utilize 5-HT receptors as either a viral entry receptor or as a co-receptor (Elphick et al., 2004; Assetta et al., 2013; Cheng et al., 2015; Cao et al., 2019). In addition, 5-HT can directly influence viral replication and yield. Depending on concentration, 5-HT has been shown to either enhance or inhibit HIV replication in T cells, while the serotonin precursor, 5-hydroxytryphan (5-HTP), can rescue IFN-g and IDO-mediated inhibition of Parainfluenza virus replication (Rabbani and Barik, 2017). Similarly, many enteric viral infections, including rotavirus, reovirus, and adenovirus stimulate the release of serotonin stores from enterochromaffin cells in the gut, resulting in enhanced viral titers, as well as serotonin-associated pathophysiological responses, including diarrhea and vomiting (Lundgren et al., 2000; Hagbom et al., 2011; Bialowas et al., 2016; Westerberg et al., 2018). Consistent with serotonin potentially facilitating host-mediated viral disease, pharmacological inhibition of serotonin reuptake by selective serotonin reuptake inhibitors (SSRI) can both suppress replication of viral pathogens and inhibit virus-mediated inflammatory disease (Zuo et al., 2012; Ulferts et al., 2013; Young et al., 2014; Medigeshi et al., 2016; Chang et al., 2017; Benkahla et al., 2018; Schneider-Schaulies and Beyersdorf, 2018; Bauer et al., 2019; Almeida et al., 2020; Calusic et al., 2021; Dechaumes et al., 2021; Meikle et al., 2021; Zimniak et al., 2021; Kummer et al., 2022).

While assessing the effect of HSV infection on cellular amino acid metabolic pathways, we unexpectedly observed upregulation of serotonin synthesis pathway genes. Based on previous studies, which indicated that 5-HT could influence pathogen replication and immune-mediated disease processes, we investigated the role of serotonin in HSV replication and its association with HSV-mediated ocular disease. The aims of the present study were to determine: (1) the effect of HSV infection on expression of serotonin-associated metabolic enzymes and transporters; (2) the consequence of HSV-induced upregulation of these pathways on *in vitro* and *in vivo* 5-HT levels; (3) the impact of extracellular 5-HT uptake and 5-HT levels on efficient HSV replication; (4) the correlation of aqueous humor 5-HT concentration with viral yield and with severity of ocular disease in a rabbit model of acute herpetic keratitis. Our findings highlight that HSV-induced upregulation of 5-HT synthesis and intracellular uptake enhances HSV replication, whereas pharmacological inhibition of these processes reduces HSV yields. Importantly, HSV-infected eyes exhibit a marked increase in aqueous humor 5-HT levels that significantly and positively correlate with severity of HSV-mediated ocular disease. These findings reveal serotonin pathways as potential novel therapeutic targets for HSV-associated ocular disease.

## Materials and Methods

### Cells, Viruses, and Reagents

Primary human corneal epithelial cells (HCEC) were obtained from Life Technologies and cultured in Human Corneal Epithelium Growth Medium with Growth Supplement Kit (Cell Applications Inc.; Sanchez et al., 2016). A549 and Vero cells were obtained from ATCC and cultured in DMEM/F-12 (Invitrogen, Carlsbad, CA, United States) supplemented with 7% FBS (Hyclone). Cells were cultured at 37°C in 5.0% CO_2_. Serotonin HCl and the SSRI inhibitor, fluoxetine-HCl, were purchased from Sigma Aldrich. The TPH inhibitor, LX-1031, was obtained from MedChemExpress (Monmouth Junction, NJ, United States). HSV-1 McKrae viral stocks were originally a gift from Dr. Jim Hill. Viral stocks were propagated in Vero cells and stored as infectious cell preparations at −80°C (Sanchez et al., 2016).

### Quantitative RT^2^ PCR Array of Amino Acid Metabolism

An amino acid metabolism I RT^2^ profiler PCR Array (Qiagen) was used to assess the expression of genes involved in amino acid metabolism (Qiagen PAHS-129Z-24). A549 cells were mock infected or infected with HSV-1 McKrae at a multiplicity of infection (MOI) of 5. Twelve hours post-infection (hpi) cells were collected, homogenized with a QIAshredder, and total RNA was purified with an AllPrep DNA/RNA Mini Kit (QiagenS) according to the manufacturer’s directions and as described previously (Garvey et al., 2014; Yanachkova et al., 2015; Nebeluk and Foster, 2020). cDNA was synthesized using the RT^2^ First Strand Kit (Qiagen). Three independent replicate samples loaded on the RT2 Profiler PCR Array were run on a Bio-Rad CFX96 Real Time System (Bio-Rad, Hercules, CA, United States) using RT^2^ SYBR Green qPCR Master Mix (Qiagen; Garvey et al., 2014; Nebeluk and Foster, 2020). Each array screened for 84 amino acid metabolism pathway-focused genes, as well as five housekeeping genes. Data were analyzed online using Qiagen analysis software (RT2 profiler PCR array data analysis V3.5) based on the ΔΔCT method with normalization of the raw data to the five housekeeping genes.

### Reverse Transcription and Quantitative PCR

Six-well plates were seeded at a density of 0.7 × 10^6^ A549 or HCEC per well. Cells were mock or HSV-1 infected (MOI 3). After a 24-h incubation period, total RNA was isolated from cells using the RNeasy Plus Mini Kit (Qiagen). RNA (500 ng) from each sample was reverse transcribed using the RT^2^ First Strand Kit (Qiagen; Garvey et al., 2014; Nebeluk and Foster, 2020). Samples were prepared for analysis with 1 μl of cDNA, 2 μl total of Forward and Reverse Primer, 10 μl of DI H_2_O, and 10 μl of iTaq Universal SYBR Green Supermix (Bio-Rad, Hercules, CA, United States) in a CFX96 Real Time System with attached C1000 Thermocycler (Bio-Rad). PCR-amplification occurred for 40 cycles using the following primer sets: Tryptophan hydroxylase 1 (*TPH1*): Forward-ACGTCGAAAGTATTTTGCGGA, Reverse-ACGGTTCCCCAGGTCTTAATC; *TPH2*: Forward-CAAAAATGACGACAAAGGCAACA, Reverse-CCTACAGTGCTTTTACCAATCCA; *DDC*: Forward-ATTCATCTGCCCTGAGTTCCG, Reverse-CCAATAGCCATTTGTGGGGAT; serotonin transporter (*SERT*): Forward-GGACAGTACCACCGAAATGGATGC, Reverse-GGTGATGTTGTCCTCGGAGAAG; and *RPLP0*: Forward-AGCCCAGAACACTGGTCTC, Reverse-ACTCAGGATTTCAATGGTGCC. The relative expression for each gene was determined by ΔΔCt analysis using RPLP0 as the housekeeping gene (Garvey et al., 2014; Nebeluk and Foster, 2020). Experiments were performed in quadruplicate.

### Cellular Protein Extraction, SDS-PAGE, and Western Blot Analysis

Cells were seeded at 0.5 × 10^6^ cells per well in a 6-well plate and grown overnight. Cells were subsequently mock- or HSV-1-infected (MOI 3) for 24 or 48 h at 37°C, as indicated. Total cell lysates were prepared in M-PER Mammalian Protein Extraction Reagent (Thermo Scientific, Rockford, IL, United States) supplemented with 0.1% SDS and 1x HALT Protease & Phosphatase Inhibitor Single-Use Cocktail (Thermo Scientific). Cell lysate preparations were freeze/thawed and centrifuged for the removal of insoluble cellular debris. Protein concentration was determined by a BCA Protein Assay (Thermo Scientific) and normalized to the lowest value. Samples were prepared for SDS-PAGE analysis in Bolt LDS Sample Buffer and Sample Reducing Agent and heated to 70°C for 15 min before being separated on Bolt 4%–12% Bis-Tris Plus gradient gels (Invitrogen). Protein was subsequently transferred to nitrocellulose membranes using an iBlot (Invitrogen), and blots were blocked with 5% nonfat dry milk (Carnation) for 1 h at room temperature before probing overnight at 40°C with the indicated primary antibody as we have described previously (Kadeppagari et al., 2012; Sanchez et al., 2012; Yu et al., 2021). The following dilutions of antibody were utilized: α-DDC: 1:1,000 (EMD Millipore, ab1569), α-TPH1: 1:1,000 (Invitrogen, PA1-777), α-TPH2: 1:3,000 (Abcam, 121,013) and, α-SERT: 1:1,000 (Abcam, ab102048). Proteins were visualized by chemiluminescence using HRP conjugated anti-rabbit (1:100,000) or anti-mouse (1:50,000) secondary antibodies and WesternBright ECL Western Blot Detection kit (Advansta, San Jose, CA, United States). To normalize protein expression, blots were subsequently stripped and re-probed with a primary antibody α-β-actin: 1:10,000 (Sigma-Aldrich) for 1 h at room temperature. All antibody dilutions and washes were performed using 0.05% Tween 20 in 1X TBS. Band density of representative immunoblots was measured by pixel intensity and normalized to corresponding levels of β-actin using ImageJ.

### Confocal Microscopy of HSV-Induced TPH1/2 Expression and Associated Serotonin Production

Cells were seeded to Grenier BioOne mclear 96-well plate for all imaging. Cell monolayers were mock- or HSV-1-infected (MOI 3) for 18 h before plates were fixed with electron microscopy grade 3% paraformaldehyde for 15 min, permeabilized with 1.0% Triton X-100, and blocked in TBS containing 5% goat sera/3% BSA for 1 h. Cells were incubated overnight with α-TPH1 (Invitrogen, PA1-777; rabbit, 1:500), α-TPH2 (Abcam, ab184505; rabbit 1:500), α-HSV-1 (Virosys, VRX-0729Y; mouse 1:1000), and/or α-serotonin (Novus, 5HT-H209; mouse 1:500) antibodies as indicated. Cells were extensively washed with TBS and subsequently incubated for 30 min with a combination of Alexafluor 488 conjugated anti-mouse IgG and Alexafluor 568 conjugated anti-rabbit IgG (ThermoFisher) diluted 1:750 in TBS-blocking buffer. Cells were again extensively washed and nuclei were counterstained with DRAQ5 (1: 5,000; Thermo Fisher, Waltham, MA, United States) before imaging. Specific immunofluorescence was examined using a Leica SP8 laser scanning confocal microscope (Leica Microsystems, Exton, PA, United States) fitted with a water immersion 63x Leica objective (1.4 numerical aperture). Individual optical sections in the *z*-axis, averaged six times, were collected in series in the different channels at 1,024 × 1,024 pixel resolution as described previously (Foster et al., 2001, 2003, 2004). Images were maximally projected to a single image, and compiled final figures were produced with Adobe Photoshop.

### Fluorescent Monoamine Uptake Potential

Cell-based fluorescent uptake assays were performed in Grenier BioOne mclear 96-well plate. Cells were plated at a density of 90,000/well and grown overnight at 37°C. Subconfluent cell monolayers were infected with HSV-1 (MOI 3) and at 24-hpi cells were incubated at 37°C for 30 min with a fluorescent surrogate monoamine for serotonin, IDT307 (Sigma-Aldrich, SML0756). Immediately before the assay, the culture medium was aspirated and cells were washed with 100 μl of uptake buffer. Cell imaging and mean fluorescent intensity (MFI) was determined by performing a well scan with Cytation1 Cell Imaging Multi-Mode Reader (Biotek, Winooski, VT, United States).

### ELISA Quantification of Serotonin

Serotonin concentrations were measured according to manufacturer’s instructions by colorimetric competitive inhibition using a serotonin ELISA kit (Enzo, Farmingdale, NY, United States). Cell supernatants from mock or HSV-1-infected cells or from extracted aqueous humor collected from HSV-1 infected rabbit eyes during various stages of the acute phase of herpetic keratitis were analyzed. Samples were diluted 1:4 in assay buffer and each sample was run in triplicate. Serotonin concentrations were determined as per the manufacturer’s instructions using a four-parameter logistic serotonin concentration standard curve generated for analysis of samples using the following formula:


$$\%Bound=\frac{Net\;OD}{Net\;Bo\;OD}x100$$

The average net OD was obtained by subtracting the average background OD. *B*_o_ is the average OD obtained for the maximum binding wells.

### Determination of Viral Yield

For examining the effects of serotonin and serotonin synthesis on HSV replication, cells in 12-well plate were grown to a subconfluent monolayer. Prior to infection with HSV-1 McKrae (MOI 5), cells were mock treated or treated as indicated with 50 μM serotonin with or without 500 nM of the TPH inhibitor, LX-1031. Similarly, the effect of serotonin uptake inhibition on HSV-1 replication was performed using 10 μM, 1 μM, or 100 nM of the SSRI, fluoxetine, in the presence or absence of 50 μM serotonin. Twenty-four hpi cells and supernatants were freeze/thawed three times to free infectious virus. Viral loads from HSV-infected rabbit eyes were determined by inserting strips of filter paper into the lower conjunctival cul de sac of rabbit eyes for 30 s to absorb tear film containing virus. Strips were placed in 1 ml DMEM/F-12 to release virus, snap frozen, and stored at −80°C. Tear film virus collections were performed just prior to harvesting aqueous humor. Viral titers from cell lysates and tear film were determined in triplicate for each sample by end point titration of virus stocks on Vero cells as described previously (Clement et al., 2011; Sanchez et al., 2012, 2016; Hill et al., 2014; Yanachkova et al., 2015).

### Retrospective Correlation Analysis of Aqueous Humor Serotonin Concentrations, Viral Replication, and Ocular Disease in a Rabbit Model of Ocular Herpetic Keratitis

The correlation of serotonin concentrations in aqueous humor with the severity of disease in a rabbit model of acute ocular herpetic keratitis was performed retrospectively from either uninfected rabbit eyes or studies that included control BSS-treated or trifluorothymidine (TFT)-treated HSV-1 infected eyes. Control and TFT treated rabbits in all studies followed an identical protocol. Prior to inoculation, the number of viral plaque-forming units (PFU) was determined with a standard plaque assay procedure with Vero cells (Sanchez et al., 2012, 2016; Hill et al., 2013, 2014). New Zealand White (NZW) rabbits (2–3 kg) obtained from RSI Robinson Services Rabbitry were inoculated with 1 × 10^4^ PFU of HSV-1 McKrae in each eye following mild scarification of the central cornea in a 4 by 4 grid pattern with a blunted 28-gauge needle. Three days after infection, rabbits were clinically scored by slit lamp biomicroscopy with 0.1% fluorescein and sorted into clinically balanced groups. Analyzed animals had been treated topically q.i.d with 50 μl of either 0.5% Carboxymethlylcellulose (CMC) BSS or 0.5% TFT in 0.5% CMC BSS. Clinical disease parameters, including epiphora/tearing, inflammatory discharge, corneal epithelium, corneal inflammation, stromal inflammation, corneal neovascularization, scleral injection/conjunctivitis, blepharitis, and fluorescent slit lamp evaluation were assessed daily in a masked fashion as previously described (Clement et al., 2011; Hill et al., 2012, 2013, 2014; Webre et al., 2012). In addition, corneal thickness was measured using a Reichert iPac Pachymeter. Virus was collected as described above on each day of clinical scoring. Aqueous humor was collected from individual eyes of rabbits randomly assigned for pathology. Matched sets of individual ocular disease clinical scores or viral titers at the day of aqueous humor collection were compared to their corresponding aqueous humor serotonin concentrations and assessed by Spearman’s and Pearson’s correlation analysis using GraphPad Prism Software.

### Statistical Analysis

All values are expressed as mean ± SEM and all data were graphed using Graph Pad Prism. Student’s unpaired *t* test was used to compare between two groups. Correlations between serotonin and individual clinical parameters or viral titers were performed using Spearman’s and Pearson’s analysis, and a 95% CI was used to determine significance of the *r* value. Statistical significance was defined as *p* < 0.05 with specific *p* values indicated in the figures.

## Results

### HSV Infection Profoundly Alters Gene Expression of Amino Acid Metabolic Pathways Associated With 5-HT Biosynthesis

**Figure 1:**
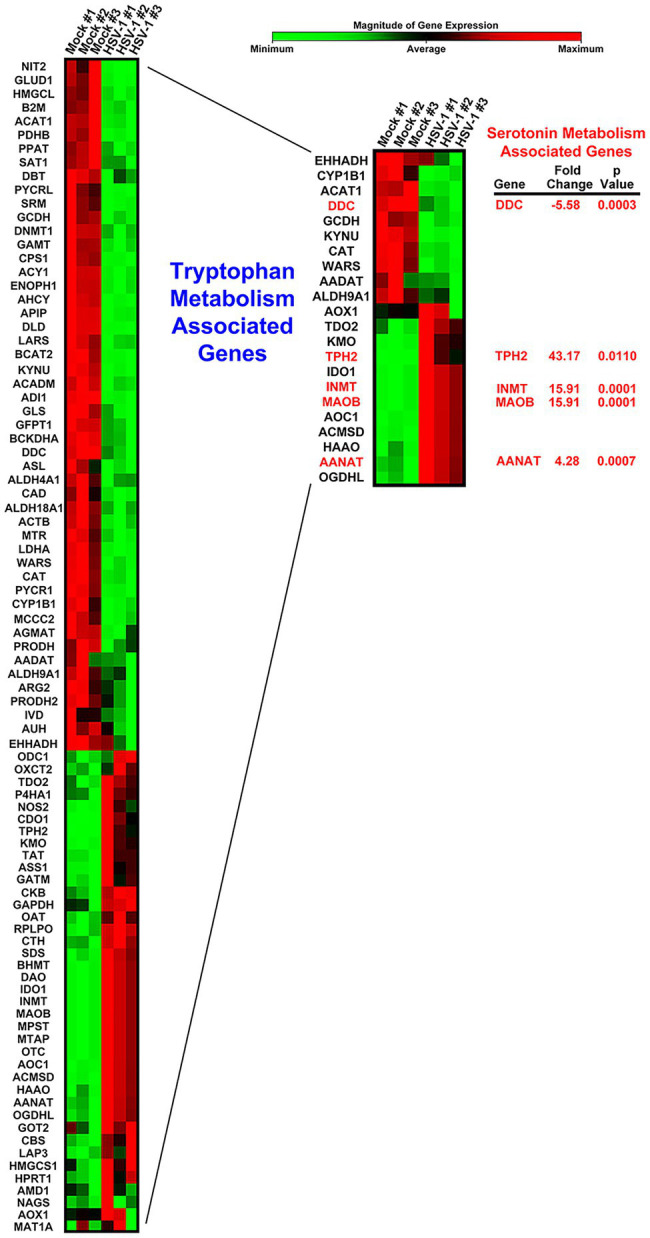
Heat map comparison of host cell amino acid metabolism gene expression following Herpes simplex virus (HSV)-1 Infection. Cells were either mock- or HSV-1-infected (MOI 5) in triplicate. Differential expression of amino acid metabolism-associated genes was determined by Qiagen RT^2^-PCR Profiler Array analysis. The resulting heat map depicting each of the genes is shown color coded by the relative magnitude of normalized gene expression. A subset of genes involved in tryptophan metabolism is displayed within the inset heatmap. Genes encoding enzymes involved in serotonin-associated metabolism are highlighted with their respective fold change and *p* values.

Viruses are absolutely dependent on host cell metabolic and biosynthetic processes for efficient replication. To determine the main amino acid associated metabolic pathways regulated by HSV-1 infection, we assessed 84 amino acid metabolism associated genes in an RT^2^ PCR Amino Acid Metabolism Array. Our results revealed a striking dichotomy between HSV-1 infected and uninfected cells, indicating that there was a stark difference in metabolic characteristics between a homeostatic and an HSV-infected cell (Figure 1; Supplementary Table 1). Grouping sets of related genes that corresponded with specific amino acid metabolic pathways revealed that many genes involved in tryptophan metabolism exhibited the highest and most significant changes in expression (Figure 1, inset; Supplementary Table 1). Within this particular cluster, serotonin metabolism-associated genes, including Aromatic L-Amino Acid Decarboxylase, also known as DOPA decarboxylase (*DDC*), *TPH2*, and Monoamine oxidase B (*MAOB*) exhibited some of the greatest fold changes (Figure 1). Intriguingly, the rate-limiting enzyme in serotonin synthesis *TPH2*, which is normally only expressed in cells of neuronal origin, was upregulated more than 40-fold following HSV-1 infection.

**Figure 2:**
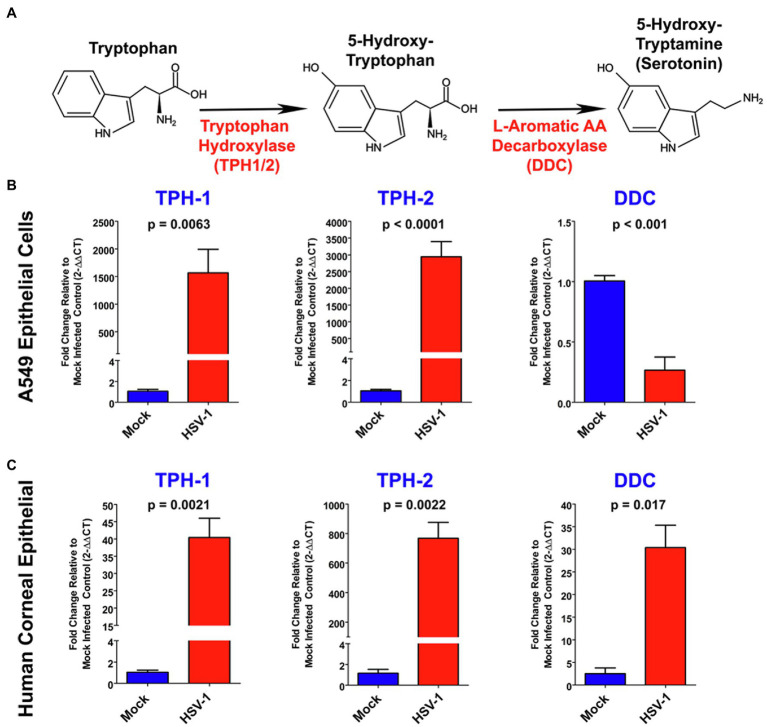
Herpes simplex virus alters expression of key genes associated with serotonin synthesis. **(A)** Schematic of the serotonin synthesis pathway indicating key enzymes involved in the synthesis of serotonin from L-Tryptophan (L-Trp). **(B,C)** Quantitative RT-PCR analysis of *TPH1*, Tryptophan hydroxylase 2 (*TPH2*), and DOPA decarboxylase (*DDC*) relative fold change in gene expression in either A549 **(B)** or primary Human Corneal Epithelial Cells (**C**; *HCEC*), comparing mock-infected to HSV-1 infected cells. Mock-infected values for each gene were set to 1, and relative fold change was determined by 2-DDCT method. Data are graphed as mean ± SEM.

Next, using targeted qRT-PCR, we confirmed that each of the genes directly involved in anabolism of serotonin from L-Trp were altered by HSV infection in both A549 and primary HCEC (Figures 2B,C, respectively). The cellular synthesis of serotonin from L-Trp is depicted in Figure 2A. The conversion of L-Trp to 5-hydroxytryptophan by either the peripheral TPH-1 or neuronal TPH-2 specific isoforms is the initial rate-limiting step in serotonin synthesis (Walther and Bader, 2003). Consistent with our PCR array results, *TPH2* was highly and significantly upregulated following HSV infection of both A549 (~3,000 fold; Figure 2B) and HCEC cells (>750 fold; Figure 2C). A relative fold increase of this magnitude would be consistent with HSV activating suppressed TPH2 gene expression following infection. HSV also significantly and highly upregulated expression of the peripheral *TPH1* gene in A549 by ~1,500 fold and HCEC cells by ~40 fold (Figures 2B,C, respectively). 5-hydroxytryptophan produced by TPH-1/2 is subsequently catalyzed by DDC to produce serotonin (5-HT, Figure 2A). Consistent with the amino acid PCR array, *DDC* gene expression was downregulated by ~70% following infection of A549 cells (Figure 2B). However, *DDC* gene expression was significantly upregulated by ~30-fold following infection of HCEC cells (Figure 2C). Taken together, these data indicate that HSV infection induces the significant upregulation of genes involved in anabolic synthesis of serotonin from L-Trp.

### HSV-Infected Cells Exhibit Increased TPH-1 and TPH-2 Protein Expression With Concomitant Increased Synthesis of 5-HT

**Figure 3:**
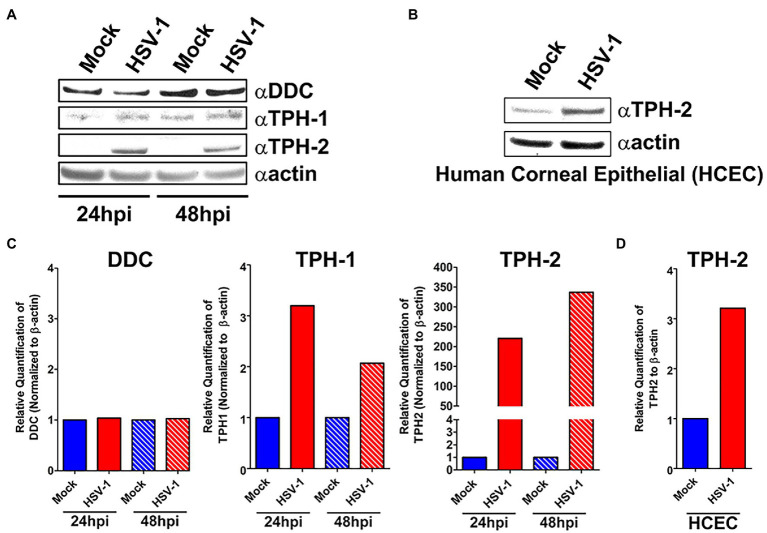
Protein expression of key serotonin synthesis enzymes is upregulated following HSV-1 Infection. **(A)** Western blot analysis of DDC, TPH-1, and TPH-2 protein expression between HSV-1-infected and mock-infected A549 cell lysates at 24 and 48 hpi. **(B)** Quantification from A549 cell western blots of relative expression of DDC, TPH-1, and TPH-2 band intensity normalized to β-actin. **(C)** Western blot analysis of TPH-2 protein expression between HSV-1-infected and mock-infected HCEC at 24 hpi. **(D)** Quantification from HCEC western blots of relative expression of TPH-2 band intensity normalized to β-actin expressed in HCEC cells.

We next assessed whether the observed HSV-induced expression of critical 5-HT synthesis genes corresponded with changes in their protein expression. Consistent with our gene expression analysis, levels of both TPH-1 (> 2-fold) and TPH-2 (> 200-fold) at 24 and 48 h increased following HSV infection of A549 cells (Figures 3A,B). Similarly, an increase in TPH-2 protein expression was observed in HSV-infected primary HCEC (Figures 3C,D). Notably, the normally neuronally restricted expression of TPH-2 was almost undetectable in the absence of HSV infection. In contrast to our RNA results, DDC protein expression was not significantly reduced at 24 or 48 h post HSV infection of A549 cells.

**Figure 4:**
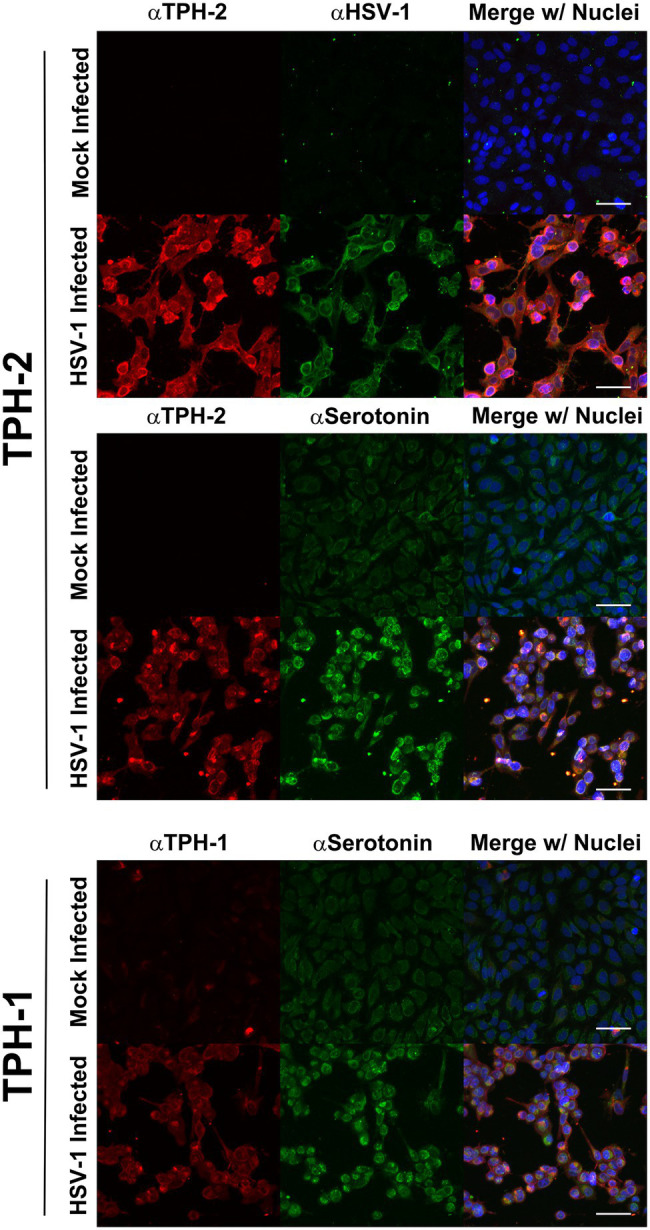
Herpes simplex virus upregulation of TPH-1 and TPH-2 expression is associated with a corresponding increase in serotonin production. Cells were mock or HSV-1 infected and at 24 h post infection, expression of TPH-2 (top and middle series of panels, αTPH2; red), TPH-1, (bottom series of panels, αTPH1; red), serotonin (middle and bottom series of panels, αSerotonin; green), or HSV-viral proteins (top series of panels, αHSV; green) was visualized by confocal immunofluorescent analysis. Cell nuclei were counterstained with DRAQ5, pseudocolored blue and channels merged.

The enhanced expression of TPH-1 and TPH-2 specifically within HSV-infected cells was further supported by confocal immunofluorescent assays (IFA; Figure 4). Congruent with our immunoblot findings and with published data on TPH, in the absence of HSV infection, cells exhibited low levels of TPH-1 (Figure 4, TPH-1; Mock Infected), while the expression of the neuron-specific TPH-2 isoform was completely absent (Figure 4, TPH-2; Mock Infected). However, in concordance with our gene expression and western analysis results, following HSV infection both TPH-1 and TPH-2 were markedly increased (Figure 4, HSV-Infected). Predictably, HSV-infected cells exhibiting increased TPH-1/2 expression displayed a corresponding increase in 5-HT levels compared to mock infected cells (Figure 4, aSerotonin). Collectively, these results indicate that HSV infection of A549 and HCEC enhanced gene and protein expression of critical 5-HT synthesis enzymes, consequently inducing a concomitant increase in cellular synthesis of 5-HT.

### Upregulation of TPH-Associated 5-HT Synthesis Enhances HSV Replication

**Figure 5:**
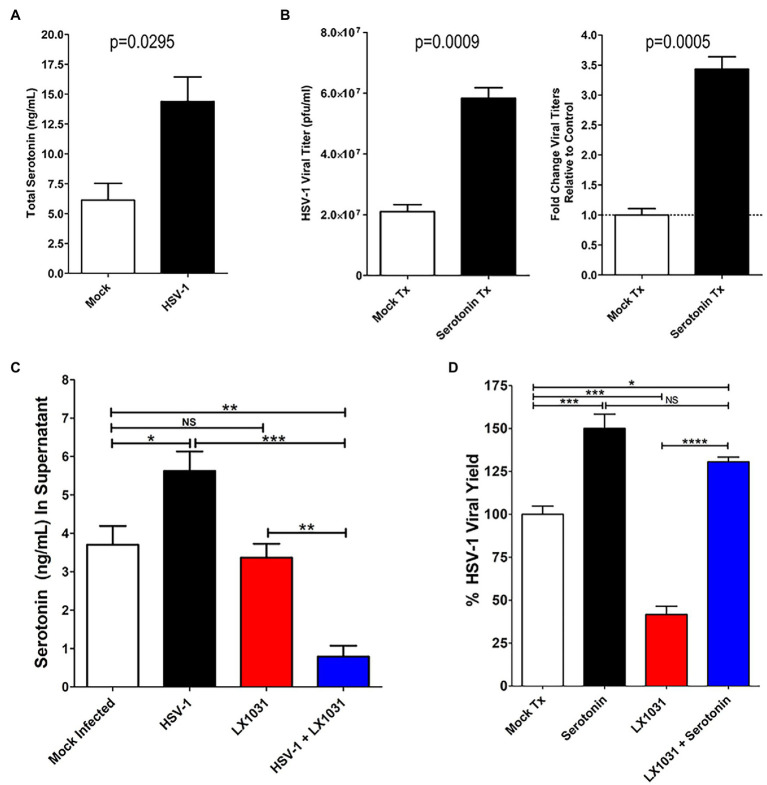
Serotonin enhanced HSV replication is suppressed by pharmacological inhibition of TPH-1/2. **(A)** ELISA determination of total intra- and extra-cellular serotonin concentrations from mock- or HSV-infected cells. **(B)** HSV-1 viral yield (left panel) and fold increase in viral yield (right panel) were determined for infected cells either mock treated or treated with 50 μM serotonin. **(C)** ELISA determination of extracellular serotonin concentration from supernatants of HSV-1 infected or mock-infected cells that had been mock treated or treated with 500 nM of the TPH inhibitor LX1031. **(D)** HSV-1 infected A549 cells were mock treated (Mock Tx), treated with 50 μM serotonin (Serotonin) and 500 nM LX1031 (LX1031), or cotreated with 500 nM LX1031 and 50 μM serotonin (LX1031 + Serotonin). Viral titers were determined using a limiting dilution plaque formation assay and were expressed as a percentage of Viral Yield Relative to Mock Treatment. Data are graphed as mean ± SEM. (^*^p < 0.05, ^**^p < 0.01, ^***^p < 0.001, ^****^p < 0.0001, and NS: not significant).

5-hydroxytryptamine production induced by HSV infection was further quantified using a colorimetric competitive inhibition ELISA. In agreement with IFA assessments, HSV-infected cells exhibited total intracellular and extracellular 5-HT concentrations that were on average more than twice that of uninfected cells (Figure 5A). 5-HT has been previously demonstrated to enhance replication of other pathogens, including viruses (Sidibe et al., 1996; Elphick et al., 2004; Hagbom et al., 2011; Westerberg et al., 2018). Therefore, the effect of serotonin synthesis on viral replication was investigated. Although physiological 5-HT levels within the plasma and sera are generally low (10 nM–1 mM), within diseased microenvironments 5-HT concentrations are greatly accentuated (>100 mM; Herr et al., 2017). Inclusion of low pathophysiological levels of serotonin (50 mM) within media of HSV-infected cells significantly enhanced HSV replication by ~3-fold compared to mock treated controls (Figure 5B).

To determine if the HSV-enhanced TPH-1/2 expression and activity was associated with serotonin-mediated increases in HSV replication, we treated cells with a peripherally restricted TPH1/2 pharmacological inhibitor, LX1031 (Camilleri, 2010, 2011) and assessed its effects on extracellular 5-HT levels (Figure 5C) and viral replication (Figure 5D). As observed with total cellular 5-HT levels, HSV infection increased 5-HT levels within infected cell supernatants (Figure 5C). Similarly, extracellular supplementation of 5-HT enhanced HSV-1 viral yields (Figure 5D). In contrast, HSV-induced increases in extracellular 5-HT were completely abrogated by LX1031-mediated inhibition of TPH-1/2 function (Figure 5C, blue bar), reducing extracellular 5-HT by >3-fold (Figure 5D). Consistent with the premise that increased TPH expression and serotonin production enhances viral replication, pharmacological inhibition of TPH1/2 with LX1031 suppressed HSV-viral yields by >50%. Intriguingly, in the absence of viral infection, LX1031 did not affect extracellular 5-HT levels (Figure 5C). This was contrasted by the marked decrease of 5-HT in extracellular supernatants of LX1031 treated HSV infected cells, suggesting that viral infection may increase cellular uptake and utilization of extracellular 5-HT pools. In agreement with this possibility, LX1031-mediated inhibition of HSV viral replication was rescued by supplementing cell supernatants with extracellular serotonin (Figure 5D, blue bar). Altogether, this data indicate that HSV upregulation of TPH enzymes facilitates serotonin synthesis and efficient viral replication within infected cells.

### HSV Increased Intracellular Uptake of 5-HT *via* Serotonin Transporters Augments Viral Replication

**Figure 6:**
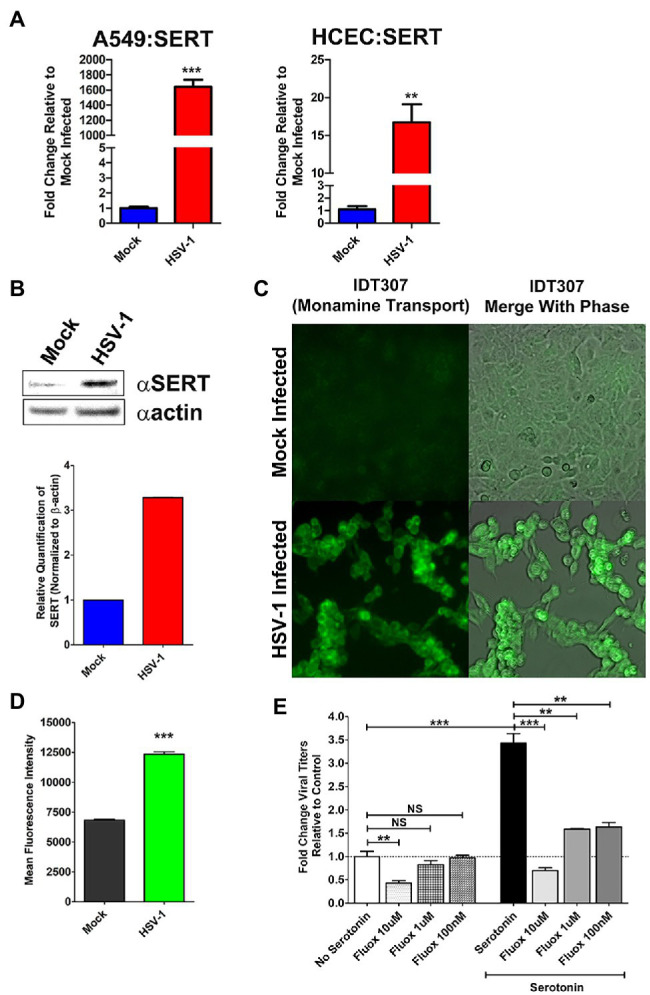
Herpes simplex virus upregulation of cellular serotonin transporter expression and serotonin uptake facilitate efficient viral replication. **(A)** Quantitative RT-PCR analysis of serotonin transporter (SERT) gene expression in mock infected vs. HSV-1-infected A549 and HCEC cells. **(B)** Western blot analysis of SERT protein expression in mock-infected vs. HSV-1 infected cells. Relative quantification was performed by normalizing band density to β-actin. **(C)** Representative fluorescent micrographs depicting enhanced uptake of the fluorescent serotonin surrogate IDT307 monoamine in mock or HSV-infected cells. **(D)** Quantitative assessment of mean fluorescent intensity (MFI) following fluorescent IDT307 in Mock or HSV-1 infected cells. **(E)** HSV-1 infected cells were treated with the serotonin reuptake inhibitor, Fluoxetine at 10 μM, 1 μM, or 100 nM in the presence of 50 μM serotonin. Relative viral yields were determined by limiting dilution plaque assays and compared to No Serotonin Controls. Data are graphed as mean ± SEM. (^**^p < 0.01, ^***^p < 0.001, and NS Not Significant).

As noted, one possible explanation for the observed decrease in extracellular serotonin following inhibition of intracellular 5-HT synthesis by LX1031 in the context of viral infection, is that HSV induces the intracellular uptake of 5-HT through active cellular monoamine transporters, such as SERT. Consistent with this hypothesis, qRT-PCR analysis of *SERT* expression revealed that there was a significant increase in serotonin transporter gene expression in both A549 (~1,600 fold) and HCEC (~16 fold) following infection (Figure 6A). Similarly, SERT protein levels were increased ~3-fold after HSV infection (Figure 6B). To ascertain if the HSV-associated upregulation of SERT expression facilitated a corresponding increase in monoamine import, the ability of HSV-infected cells to uptake a fluorescent monoamine surrogate of serotonin, IDT307 (Beikmann et al., 2013; Tomlinson et al., 2021), was assessed relative to mock-infected cells (Figures 6C,D). As expected, cells infected with HSV exhibited significantly greater MFI compared to mock-infected cells, indicating an enhanced ability to uptake the serotonin surrogate, IDT307.

Finally, we assessed the effect of pharmacological inhibition of cellular 5-HT uptake on HSV replication. FCS supplemented media contain physiological levels of serotonin. In the absence of pathophysiological levels of serotonin, HSV replication was significantly impaired at the highest tested concentration of fluoxetine, a SSRI (Figure 6E; Wong et al., 2005). As shown earlier, pathophysiological supplementation of extracellular 5-HT enhanced viral yields compared to viral infections cultured in media not supplemented with additional 5-HT (Figure 6E). In contrast to media with physiological levels of serotonin, infected cells co-treated with pathophysiological levels of 5-HT and different doses of fluoxetine-exhibited significantly reduced viral titers across all concentrations of fluoxetine tested (Figure 6D). Unlike our observations with LX1031 treatment, due to fluoxetine’s differing mechanism of action, the reduction in viral titers could not be rescued by further supplementation with excess extracellular serotonin. Collectively, this data indicate that HSV-infected cells augment TPH-mediated 5-HT synthesis through increased uptake of extracellular 5-HT, which facilitates optimal viral replication.

### Increased 5-HT Levels in Aqueous Humor of HSV Infected Eyes Correlate With Infectious Viral Load in a Rabbit Model of Acute Herpes Keratitis

**Figure 7:**
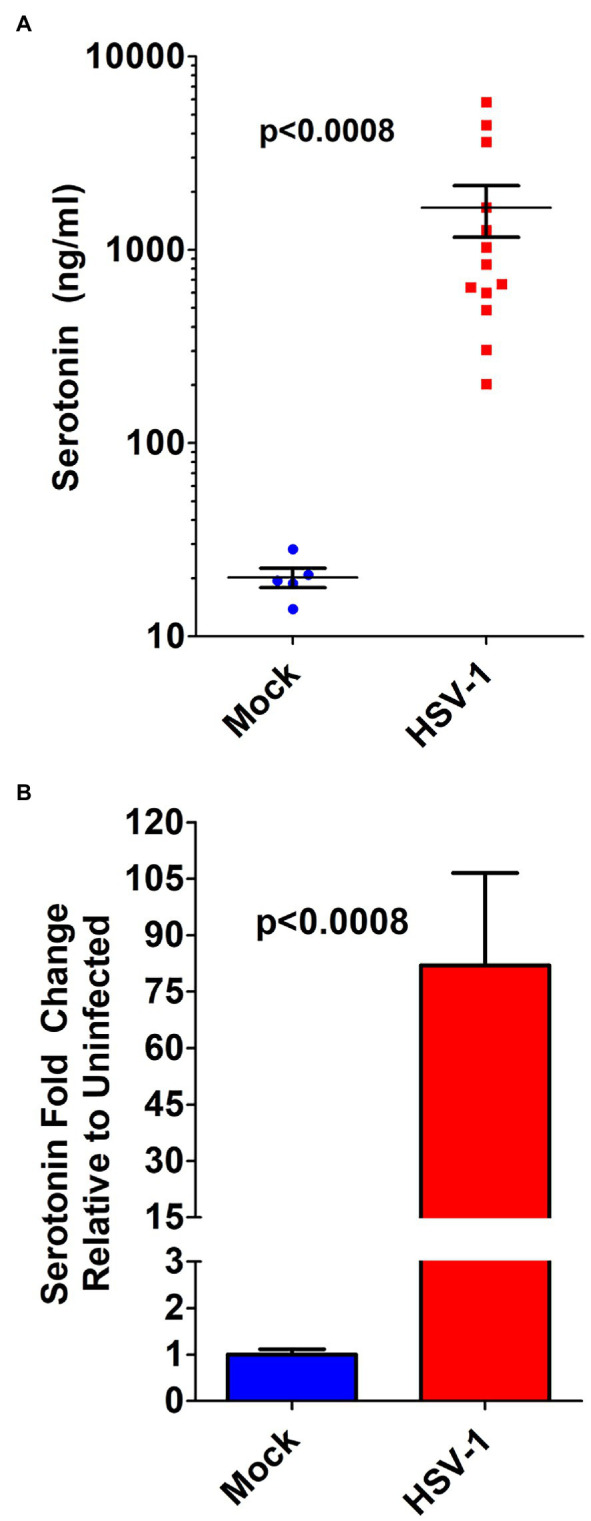
Serotonin concentration in the aqueous humor of HSV-infected eyes is markedly increased in a rabbit model of acute herpes keratitis. **(A)** ELISA determination of serotonin concentration from the aqueous humor of individual rabbit eyes that was mock- or HSV-1-infected. **(B)** Fold change in serotonin concentration in the aqueous humor of rabbits mock infected or HSV-1 infected. Data graphed as mean ± SEM.

To establish the potential *in vivo* relevance of our *in vitro* cellular findings, we retrospectively analyzed recently banked AH from a rabbit model of acute herpetic keratitis and evaluated peripheral AH 5-HT concentrations from infected and uninfected eyes. The 5-HT levels in the AH from HSV-infected rabbit eyes were on average almost two orders of magnitude increased compared to mock infected eyes (Figures 7A,B). Although mock-infected AH contained a fairly consistent ~20 ng/ml 5-HT, the AH from HSV-infected eyes exhibited a range of concentrations from ~200 to ~5,800 ng/ml.

**Figure 8:**
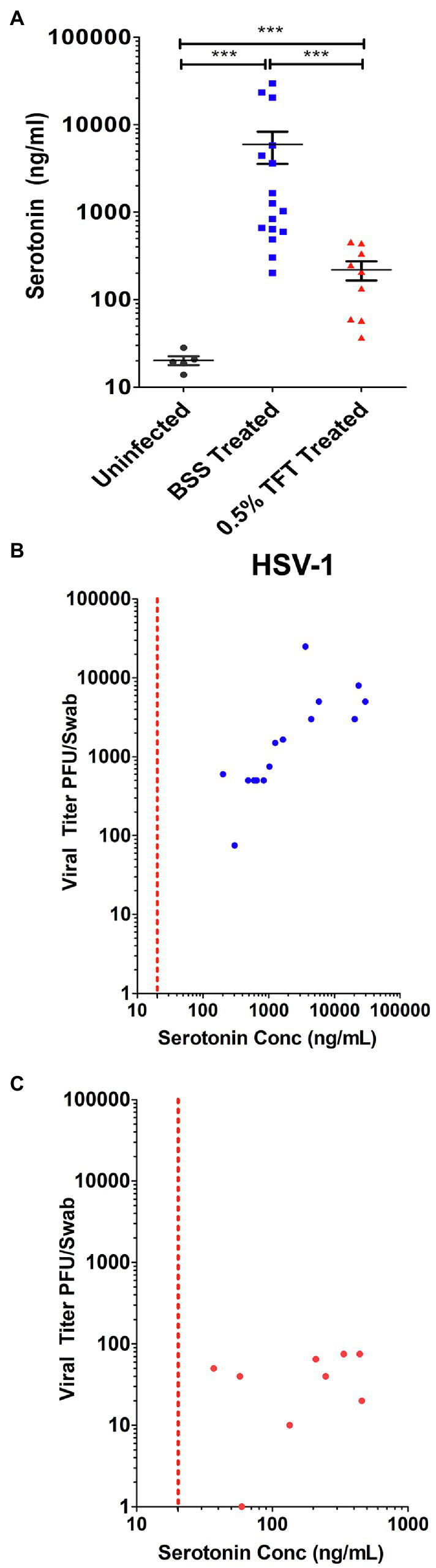
Ocular serotonin concentrations correlate with levels of infectious HSV-1 shed in rabbit eye tear films. **(A)** Serotonin concentration was measured by ELISA from the aqueous humor of rabbits that were mock- or HSV-1-infected and subsequently had their eyes treated topically with either the ocular antiherpetic 0.5% TFT or a BSS Control. **(B,C)** Correlation of FIGURE 8 | AH serotonin concentrations with infectious HSV-1 in tear films from either BSS treated control eyes **(B)** or eyes treated with the antiviral 0.5% TFT **(C)**. AH serotonin concentrations were compared to corresponding infectious HSV-1 viral titers isolated from tear film swabs. Viral titers were determined by limiting dilution plaque assay on Vero. The Spearman’s correlation coefficient, reflecting the rank order correlation between the identified two variables (denoted by *r*), and the significance of those associations (*p* value, *p*) are indicated within the text. ^***^p < 0.001.

We further compared 5-HT levels from AH samples obtained during a study that included an antiviral treatment arm. Infected rabbit eyes, which were treated with 0.5% TFT to suppress HSV replication, exhibited a smaller range of 5HT concentrations that were significantly decreased compared to 5-HT levels in AH from control BSS treated infected eyes. However, 5-HT levels were still significantly higher than mock infected controls (Figure 8A). To determine if the observed increased 5-HT levels in AH correlated with viral yield, we compared the levels of AH 5-HT with the amount of infectious HSV virus isolated from corresponding swabs of tear film. Consistent with *in vitro* data indicating that serotonin levels increased viral yields, as 5-HT levels increased within the AH of infected eyes, there was a concomitant increase in infectious virus isolated from tear film (Figure 8B) that exhibited a significant, strong, and positive Spearman’s correlation (*r* = 0.8656; *p* < 0.0001). By contrast, eyes treated with 0.5% TFT exhibited both lower viral titers and correspondingly lower 5-HT concentrations (Figure 8C). Because TFT directly impacts infectious viral production irrespective and independent of 5-HT concentrations, as expected, 5-HT levels did not correlate with viral titer in eyes treated with TFT (Spearman’s correlation; *r* = 0.2941; *p* = 0.4366). Collectively, these data indicate that like our *in vitro* cellular findings, HSV infection greatly enhances 5-HT levels within the microenvironment of the infected eye.

### HSV-Induced Ocular Clinical Disease Severity Has a Strong Positive Correlation With AH Serotonin Levels

**Figure 9:**
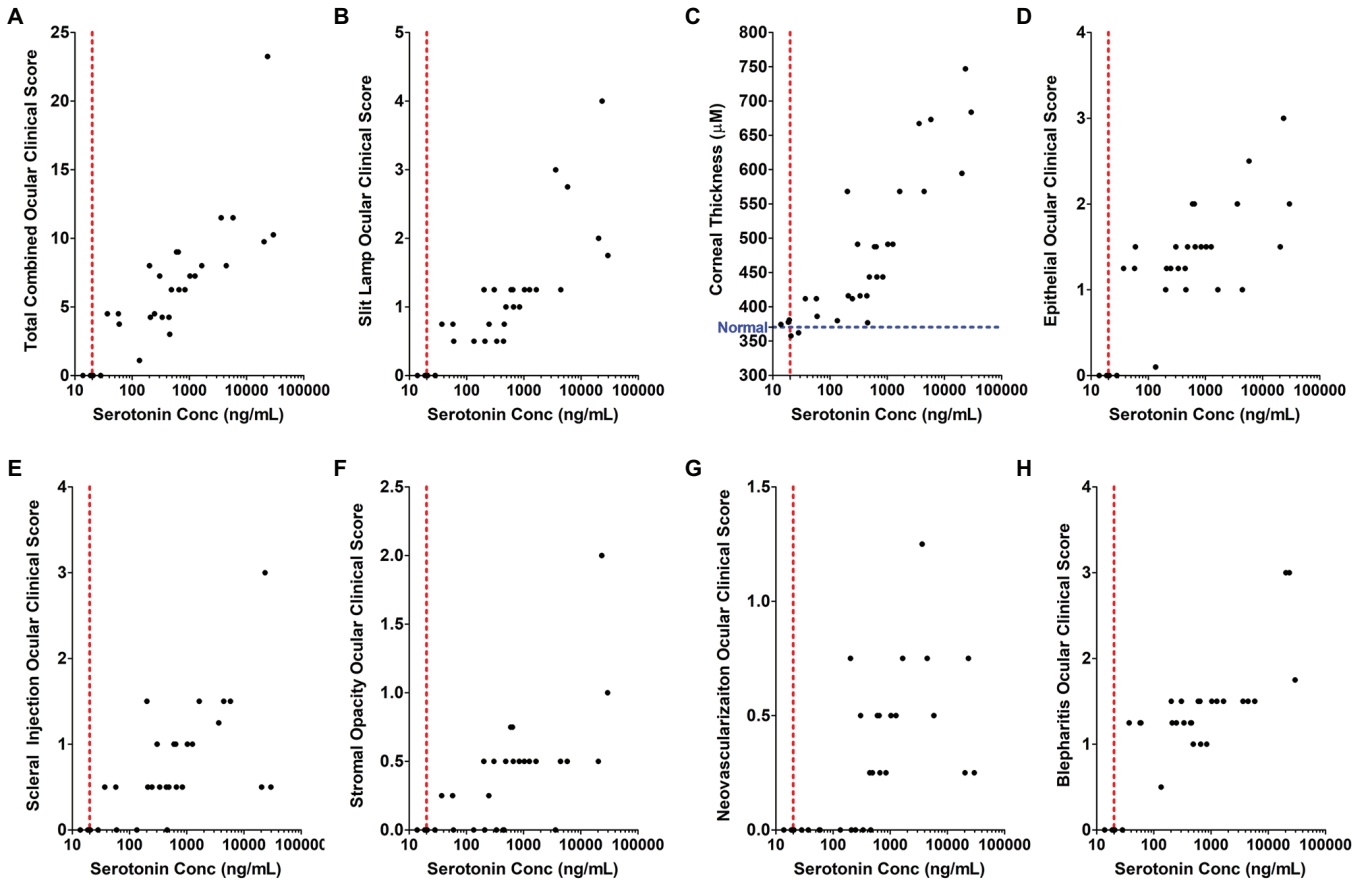
The concentration of serotonin in the aqueous humor positively and significantly correlates with individual inflammation-associated ocular disease parameters in a rabbit eye model of acute herpetic keratitis. **(A–H)** Correlation analysis was performed comparing serotonin concentration in the AH of HSV-infected rabbit eyes relative to its corresponding clinical score for each of the indicated clinical assessment parameters. **(A)** Combined total clinical ocular scores; **(B)** Fluorescent slit lamp biomicroscopy; **(C)** Corneal thickness measured by Pachymeter (Blue line indicates the average normal thickness of an uninfected cornea); **(D)** Epithelial damage and inflammation; **(E)** Scleral injection; **(F)** Stromal opacity; **(G)** Corneal neovascularization; and **(H)** Blepharitis. The Spearman’s correlation coefficient (denoted by *r*), which reflects the rank order correlation between the identified two variables, and the Pearson’s correlation coefficient (denoted by *r*), which measures the strength of the linear relationship between the two variables are indicated within Table 1 with the corresponding significance of those associations (*p* value, *p*).

Although self-limiting in nature, HSV ocular infections lead to dendritic or geographic ulceration of the cornea, destruction of the corneal epithelium with corneal thickening, and inflammation-mediated disease manifestations. The recent association of peripheral 5-HT with inflammatory disease processes led us to evaluate the correlation between HSV-induced 5-HT production and severity of HSV-associated clinical disease. Therefore, clinical disease assessments that were evaluated on the day of AH collection were paired with their respective AH 5-HT levels and analyzed by Spearman’s and Pearson’s analysis to determine the degree of correlation (Figure 9; Table 1). Overall, the total combined clinical scores and the majority of individual clinical disease assessments exhibited a significant, strong, and positive correlation with AH 5-HT levels. These assessments included both defined, but subjective, observer-based scores, as well as an objective direct measure of corneal thickness. Although most clinical assessments exhibited a significant correlation with 5-HT levels, albeit to different extents, neovascularization did not exhibit a significant Pearson’s correlation (*r* = 0.2315; *p* = 0.1092). It was noted, however, that the extent of corneal neovascularization induced by HSV-infection in this acute model was minimal on the scoring scales (Figure 9G; Table 1). Collectively, our data demonstrate that 5-HT levels are highly elevated in HSV-infected eyes and these levels strongly and positively correlate with efficient infectious virus production and severity of ocular disease.

**Table 1:** Spearman’s and Pearson’s correlation coefficients, including *r* and *p* values of each clinical disease assessment compared to AH Serotonin Levels from Figure 9.

Clinical parameter	Correlation	*r*	*p* value
Total combined parameters	Pearson	0.6457	<0.0001
	Spearman	0.8668	<0.0001
Slit lamp	Pearson	0.6327	<0.0001
	Spearman	0.8828	<0.0001
Corneal thickness	Pearson	0.7264	<0.0001
	Spearman	0.875	<0.0001
Epithelial	Pearson	0.486	0.0032
	Spearman	0.7348	<0.0001
Scleral total injection	Pearson	0.3899	0.0166
	Spearman	0.7029	<0.0001
Stromal opacity	Pearson	0.6696	<0.0001
	Spearman	0.7021	<0.0001
Blepheritis	Pearson	0.6231	<0.0001
	Spearman	0.7971	<0.0001
Neovascularization	Pearson	0.2315	0.1092
	Spearman	0.7417	< 0.0001
Epiphora	Pearson	0.9127	<0.0001
	Spearman	0.8624	<0.0001

## Discussion

In this study, our findings highlight that HSV significantly alters cellular serotonin-associated metabolic pathways resulting in an increased production of 5-HT. We also identified two mechanisms that HSV utilizes to obtain 5-HT, which when inhibited, suppressed infectious viral yields: upregulation of 5-HT synthesis pathways and uptake of 5-HT into the cell from the extracellular environment through SERT. Correspondingly, in *in vitro* studies, 5-HT supplementation augmented production of infectious viruses, whereas pharmacological inhibition of either TPH-mediated 5-HT production or cellular uptake suppressed viral yields. *In vivo*, levels of 5-HT within the aqueous humor of HSV-infected eyes significantly and positively correlated both with the amount of infectious virus isolated from infected eyes and the severity of HSV-mediated ocular disease. Although our *in vivo* assessments were limited by the retrospective and correlative nature of banked sample assessment, due to the strong correlations observed, we are confident that our findings reveal that HSV alters critical serotonin metabolic pathways that are associated with HSV-mediated ocular disease development. Therefore, our findings clearly support further mechanistic and pharmacological targeting of these serotonin-associated metabolic pathways to clearly establish its specific roles in HSV-associated ocular disease development.

As obligate intracellular pathogens, viruses must alter host cell metabolism both to provide resources necessary for efficient viral replication and to aid in evasion of cell-intrinsic, innate, and adaptive host immune responses (Maynard et al., 2010; Purdy and Luftig, 2019; Thaker et al., 2019). Amino acid metabolic pathways are essential for cellular processes that are required for efficient viral replication and immune responses to pathogens. Consequently, viruses are dependent on either amino acid availability or metabolism for their optimal replication (Mehraj and Routy, 2015; Sanchez et al., 2016; Raniga and Liang, 2018; Thaker et al., 2019; Costantini et al., 2020). It was therefore not unexpected that compared to uninfected cells, HSV-infected cells exhibited diametrically opposed expression of amino acid metabolism-associated genes. However, we did not anticipate that serotonin metabolism-associated genes would be overrepresented as the most highly and significantly affected genes.

In HSV-infected cells, we unexpectedly observed transcriptional activation of genes associated with the rate-limiting enzymes in serotonin synthesis, *TPH1* and *TPH2*. Although *TPH1* can be expressed at low levels within many cell types, *TPH2* expression is normally neuronally restricted and is suppressed in all non-neuronal cells through the repressive complex RE-1 Silencer of Transcription/Neuron Restrictive Silencing Factor (REST/NRSF; Patel et al., 2007). Similarly, upon entry into the nucleus, HSV genomes are immediately bound by repressive histones and cellular repressors, including the REST/CoREST repressor complex. Therefore, HSV gene expression is dependent on coordinate and sequential derepression of viral genes within its genome to initiate successful viral replication (Roizman, 2011). As such, to facilitate expression of its own genes and replication in non-neuronal cells, HSV has dedicated several viral proteins to disrupt the suppressive effects of the REST/NRSF repressor complex (Roizman, 2011). In addition, HSV diverts a component of the repressive complex, lysine-specific demethylase 1 (LSD1), to demethylate suppressive epigenetic marks and thereby activate transcription (Roizman, 2011; Hill et al., 2014). The effects of HSV on these processes may therefore facilitate expression of *TPH2* in peripheral cells *via* derepression and activation of the *TPH2* promoter. Intriguingly, other viruses regulate these same processes to enable their replication and therefore, may also induce *TPH2* expression and upregulation of 5-HT synthesis (Guan et al., 2009; Sakane et al., 2011). For example, Adenovirus, another ocular viral pathogen, similarly inactivates REST/NRSF repressive functions through expression of E1A, thereby inducing normally suppressed neuron-associated gene expression (Guan et al., 2009). Consistent with this association, we have observed similar upregulation of *TPH2* expression in Adenovirus infected epithelial cells (unpublished observations). Therefore, viral induction of TPH enzymes and 5-HT synthesis may be a common theme among numerous viral pathogens to enable their efficient replication and consequently may also contribute to disease development.

Western blot analysis confirmed that expression of both TPH-1 and TPH-2 enzymes were upregulated following HSV infection. As denoted in Figure 2, TPH catalyzes the hydroxylation of L-Trp to 5-HTP, the precursor of serotonin. It is possible that HSV-induces upregulation of TPH enzymes to shunt limited L-Trp stores toward 5-HT metabolic pathways and away from the IDO-initiated production of antiviral kynurenine metabolites that have been shown to reduce viral transcription and translation (Mehraj and Routy, 2015; Rabbani and Barik, 2017; Raniga and Liang, 2018). However, we strongly believe that virally induced 5-HT production is not just to sequester L-Trp away from the kynurenine pathway. As we have demonstrated here, and others have shown, supplementation with serotonin can enhance viral replication independent of L-Trp availability. In addition, 5-HTP supplementation can rescue IFN-g and IDO-mediated inhibition of Parainfluenza virus replication (Rabbani and Barik, 2017). Taken together, these results suggest that 5-HT and/or its metabolites function to actively enhance viral replication, rather than simply shunting L-Trp away from the kynurenine pathway.

Subsequent decarboxylation of 5-HTP by aromatic L-amino acid decarboxylase (DDC; Figure 2), ultimately results in generation of 5-HT. Transcriptionally, we did note differences in DDC transcript levels between A549, which exhibited decreased DDC expression in the RT^2^ Profiler array and qRT-PCR assessments, vs. primary HCEC cells, which exhibited increased DDC expression. Despite DDC transcripts being decreased in HSV-infected A549 cells, there was no corresponding change in DDC protein levels at 24 or 48 h post infection. Furthermore, the levels of DDC did not appear to be limiting, enhanced 5-HT production from HSV infected cells was observed. Interestingly, DDC overexpression has been shown to negatively affect replication of flaviviruses, while DDC enzymatic inhibition enhanced viral replication. DDC inhibition of flavivirus replication is reported to be through interaction and inhibition of phosphatidylinositol-3-kinase (PI3K)/AKT signaling pathways, which regulates key cellular processes vital to efficient replication of viruses (Frakolaki et al., 2019). HSV is known to employ multiple mechanisms to activate PI3-kinase/AKT pathways that enhance both cell survival and HSV replication (Liu and Cohen, 2015). Similarly, 5-HT engagement with its cognate cell surface receptors strongly activates PI3K-associated signaling pathways, thereby providing one potential mechanism by which virally induced 5-HT production could enhance viral replication (Nichols and Nichols, 2008).

Herpes simplex virus-induced upregulation of 5-HT metabolic pathways resulted in a concomitant increase of 5-HT in both *in vitro* cell assays and an *in vivo* rabbit model of HSV-associated eye disease. *In vitro*, 5-HT augmented production of infectious virus, while pharmacological inhibition of TPH enzymatic activity suppressed viral yields by ~50%. Importantly, *in vivo* levels of 5-HT within the AH of HSV-infected rabbit eyes significantly and positively correlated with the amount of infectious virus present within the corresponding tear film. As noted earlier, 5-HT production may also be involved in enhancing replication of other viral pathogens, including HIV and parainfluenza virus, as well as many enteric viral infections, including rotavirus, reovirus, and adenovirus, which exhibit enhanced viral titers upon release of serotonin stores from enterochromaffin cells of the gut (Sidibe et al., 1996; Elphick et al., 2004; Koh and Bertoletti, 2009; Hagbom et al., 2011; Cheng et al., 2015; Bialowas et al., 2016; Rabbani and Barik, 2017; Westerberg et al., 2018; Cao et al., 2019). Currently, the peripherally restricted TPH inhibitor utilized in this study, LX1031, is being investigated clinically for use in treating irritable bowel syndrome (IBS), which is characterized by excess 5-HT (Camilleri, 2010; Brown et al., 2011; Camilleri, 2011). Our and others’ data suggest that targeting excess peripheral 5-HT production, by small molecules like LX1031, may reduce infectious virus production and possibly host immune-mediated disease in non-CNS viral infections. It is important to note that the peripherally restricted nature of LX1031 abrogates the deleterious issues linked with inhibition of CNS-associated serotonin synthesis. However, the inability of LX1031 to enter the CNS similarly restricts its potential use to non-CNS HSV-associated disease manifestations, an important caveat for a neurotropic virus like HSV-1. In this regard, the role of serotonin either in facilitating HSV replication within neurons or in latency-reactivation, which contributes to chronic ocular disease development, is an area of future research interest.

Herpes simplex virus was also demonstrated to upregulate SERT expression and to stimulate uptake of a serotonin surrogate monoamine. SSRI-mediated inhibition of 5-HT cellular uptake suppressed pathophysiological 5-HT mediated enhancement of HSV-replication by >3-fold, indicating that active uptake and subsequent metabolic processing of 5-HT may be involved in the viral replication enhancing abilities of serotonin. Intracellular 5-HT regulates cell redox potential during its monoamine oxidase-initiated metabolism to 5-hydroxyindole acetate. Breakdown of serotonin through this pathway results in NADH/NADPH production, key molecules in both combatting virus-killing oxidative assaults and production of metabolic products and energy reserve required for efficient viral replication (Boccuto et al., 2013; Yano et al., 2015; Fiore and Murray, 2021; Groth et al., 2021). In agreement with this idea, we observed a significant ~16-fold increase in expression of *MAOB* in the RT^2^ Profiler Array, suggesting that metabolic products downstream of serotonin may provide an intracellular environment conducive for efficient viral replication. A recent publication has suggested that SSRI’s do not affect the replication of HSV-1 (Zimniak et al., 2021). As noted in our studies, at physiological concentrations of serotonin, only higher, but still pharmacologically relevant, doses of fluoxetine affected the yield of infectious HSV virions. Furthermore, the HSV assays performed by Zimniak et al. differed from our assessments in that they analyzed the number of GFP positive cells present following viral entry of a GFP expressing HSV and not generation of infectious progeny virions as noted here. Taken together, the data suggest that viral entry is not a likely mechanism by which SSRI-mediated inhibition of serotonin uptake impairs production of infectious progeny virions. However, as also noted by Zimniak et al., the ability of SSRI’s to inhibit viral replication is not unique to our findings for HSV. SSRI’s have been shown to suppress efficient replication of many viruses, including Enteroviruses, Dengue, Coxsackie virus, HIV, and SARS-CoV-2, suggesting that targeting 5-HT associated pathways may be a viable means of disrupting replication of a broad range of viral pathogens (Benton et al., 2010; Zuo et al., 2012; Ulferts et al., 2013; Young et al., 2014; Alidjinou et al., 2015; Medigeshi et al., 2016; Benkahla et al., 2018; Bauer et al., 2019; Clelland et al., 2021; Dechaumes et al., 2021; Zimniak et al., 2021).

Although 5-HT levels were significantly increased by ~2 fold in cell culture assays, during HSV ocular infection, AH 5-HT levels were found to be enhanced by almost 100-fold on average. Elevated 5-HT levels within the AH are not likely to be solely derived from HSV-induced serotonin synthesis, since 5-HT levels were likewise highly elevated in eyes topically treated with antivirals. In the periphery, platelets and mast cells store large amounts of 5-HT that upon tissue injury is released to initiate wound healing processes and immune cell infiltration (Herr et al., 2017; Schoenichen et al., 2019; Lin and Hu, 2021). In immune privileged tissues like the eye, these processes can contribute to disease development. 5-HT is also being increasingly recognized for its roles in chronic inflammation-associated diseases. Specifically, in T cells, which are in part responsible for the immunopathogenesis associated with HSV infections of the eye, 5-HT functions as an accessory factor that enhances disease-promoting T cell activation and proliferation (Leon-Ponte et al., 2007). Congruently, in a rabbit model of acute herpes keratitis, we observed a strong, positive, and significant correlation between 5-HT levels in AH and many inflammation-related clinical assessments of ocular disease severity. Although excess 5-HT in tears has been associated with inflammatory dry eye, little is known about the direct role of 5-HT in other ocular diseases (Chhadva et al., 2015). However, peripheral 5-HT levels are elevated in diabetic and hypertensive patients, two conditions which contribute to a myriad of ocular diseases. In addition, prolonged SSRI use, which enhances 5-HT signaling through cell surface receptors, has been linked to ocular hypertension, decreased tear production, and damage to ocular surface cells (Acan and Kurtgoz, 2017).

In conclusion, our data reveal that HSV infection highly upregulates 5-HT-associated metabolic pathways that culminates in the increased synthesis and intracellular uptake of 5-HT. Upregulation of these processes *in vitro* and *in vivo* correlates with enhanced viral replication and infectious viral yields, as well as *in vivo* with severity of inflammation-related ocular disease assessments. Importantly, targeting 5-HT associated metabolic pathways *in vitro via* pharmacological inhibition of either 5-HT synthesis *via* TPH-1/2 inhibitors or intracellular uptake *via* SSRIs suppressed efficient HSV-1 replication. From these and other studies, it can be speculated that modulation of 5-HT associated pathways either *via* these identified targets or through 5-HT receptor agonism/antagonism may be exploited therapeutically to ameliorate viral- and immune-mediated ocular diseases. Indeed, several 5-HT receptor agonists and antagonists have been shown to have anti-inflammatory activity (Tullis et al., 2015; Flanagan and Nichols, 2018; Flanagan et al., 2019a,b; Yu et al., 2021). Given the current pandemic, it is notable that severe COVID-19 patients, who also can present with ocular manifestations, exhibit elevated peripheral serotonin levels. Recent reports also indicate that patients on SSRIs have a lower likelihood of clinical deterioration and experience reduced SARS-CoV-2 viral loads compared to placebo patients (Lenze et al., 2020; Calusic et al., 2021; Clelland et al., 2021; Ha et al., 2021; Schloer et al., 2021). Collectively, these findings begin to illustrate that 5-HT metabolic and signaling pathways are an underappreciated and underexplored area with the potential to develop targeted broadly active therapeutics against a multitude of viral- and inflammation-mediated diseases.

## Data Availability Statement

The original contributions presented in the study are included in the article/**Supplementary Material**; further inquiries can be directed to the corresponding author/s.

## Ethics Statement

The animal study was reviewed and approved by Louisiana Health Sciences Center, New Orleans, Institutional Animal Care and Use Committee.

## Author Contributions

TF: conceptualization, funding acquisition, project administration, and supervision. DB, MS-P, CN, and TF: experimental methodology and data acquisition. DB and TF: data compilation, analysis, and visualization. DB and TF: writing original draft. All authors contributed to the article and approved the submitted version.

## Funding

This work was supported by Eleusis Benefit Corporation, a Louisiana LIFT2 Award (HSCNO-2017-LIFT-001) and by the National Institutes of Health and National Institute of General Medical Sciences P30GM106392, as well as U54GM104940 through the Louisiana Clinical and Translational Science Center. In addition, this work was supported in part by R01AI112402 from the National Institute of Allergy and Infectious Diseases. The content is solely the responsibility of the authors and does not necessarily represent the official views of the National Institute of General Medical Sciences, the National Institute of Allergy and Infectious Diseases, or the National Institutes of Health.

## Conflict of Interest

The authors declare that TF and CN received funding for this and related studies from Eleusis Corporation. The funder was not involved in the study design, collection, analysis, interpretation of data, the writing of this article, or the decision to submit it for publication. TF and CN have pending intellectual property through the LSU Health Sciences Center that is currently licensed by Eleusis Corporation and serve as members of one of Eleusis’ scientific advisory boards.

The remaining authors declare that the research was conducted in the absence of any commercial or financial relationships that could be construed as a potential conflict of interest.

## Publisher’s Note

All claims expressed in this article are solely those of the authors and do not necessarily represent those of their affiliated organizations, or those of the publisher, the editors and the reviewers. Any product that may be evaluated in this article, or claim that may be made by its manufacturer, is not guaranteed or endorsed by the publisher.

## Supplementary Material

Supplementary Table 1 RT^2^-PCR Amino Acid Metabolism Array Gene Expression Analysis of Mock versus HSV Infected Cells. Blue: Tryptophan Metabolism Associated Genes; Red: Serotonin Metabolism Associated Genes.
